# New data on the distribution and microphotographs of radiolarians in the bottom surface sediments of the North Pacific and Bering Sea obtained within the KALMAR II and INOPEX projects

**DOI:** 10.1016/j.dib.2019.104448

**Published:** 2019-08-29

**Authors:** Alexander Matul, Andrea Abelmann, Rainer Gersonde

**Affiliations:** a Shirshov Institute of Oceanology, Russian Academy of Sciences, Department of Marine Geology, Nahimovskiy Prospekt 36, 117997, Moscow, Russia; b Alfred Wegener Institute Helmholtz Center for Polar and Marine Research, Department of Geosciences, Wegener-Haus (D) Am Alten Hafen 26, 27568, Bremerhaven, Germany

**Keywords:** North Pacific, Modern marine environments, Marine micropaleontology, Radiolarian analysis

## Abstract

We present an extensive dataset on the modern radiolarian distribution in new samples of the surface sediments from the North Pacific and Bering Sea north of 38°N. Samples came from the RV Sonne cruises SO201-2 and SO202-1 in 2009 within the KALMAR II and INOPEX projects (Dullo et al., 2009, Gersonde, 2012). We have analyzed 46 surface sediment samples from the multicorers following the standard laboratory treatment, preparation of the micropaleontological slides, and counting of the radiolarian tests under the microscope (Abelmann, 1988, Zielinski et al., 1998). List of species consists of two hundred eight radiolarian taxa. During the routine counting, we made the microphotographs of radiolarians. Our dataset consists of three data files: 1) coordinates of stations, 2) list of the radiolarian taxa with microphotographs, 3) data on the raw counts of the radiolarian tests per 1 slide, and calculated total radiolarian abundances and taxa percentages.

**Table:** Specifications Table

Subject area	*Marine environments, marine micropaleontology.*
Specific subject area	*Radiolarian analysis.*
Type of data	*Tables, microphotograph images.*
How data was acquired	*Sediment sampling during the marine expedition, laboratory treatment of the sediment samples, preparation of the micropaleontological slides, analysis of slides under the microscope.*
Data format	*Raw (radiolarian counts* per *one micropaleontological slide and calculated total radiolarian abundances and species percentages). Microphotographs of radiolarians saved in TIFF format.*
Parameters for data collection	*Laboratory treatment of the marine sediment samples with the hydrogen peroxide and sodium pyrophosphate, preparation of the micropaleontological slides with Mountex mounting media.*
Description of data collection	*Quantitative radiolarian analysis under the microscope based on the counting of at least 350–400 radiolarian specimens* per *one micropaleontological slide.*
Data source location	*1) Shirshov Institute of Oceanology (SIO), Moscow, Russia.* *2) Alfred Wegener Institute Helmholtz Center for Polar and Marine Research (AWI), Bremerhaven, Germany.*
Data accessibility	*Data is with this article.*
Related research article	[1], [2].

**Table:** 

**Value of the data** • To add new micropaleontological information to the existing regional modern databases • To provide detailed quantitative data on the typical radiolarians as a reference dataset for the reconstructions of the North Pacific paleoenvironments • To specify modern biogeography and biodiversity in the North Pacific • To extend knowledge about the modern marine ecosystems in the high-latitude cold-water areas

## 1 Data

We present three data files in this article.

**Fig. 1:**
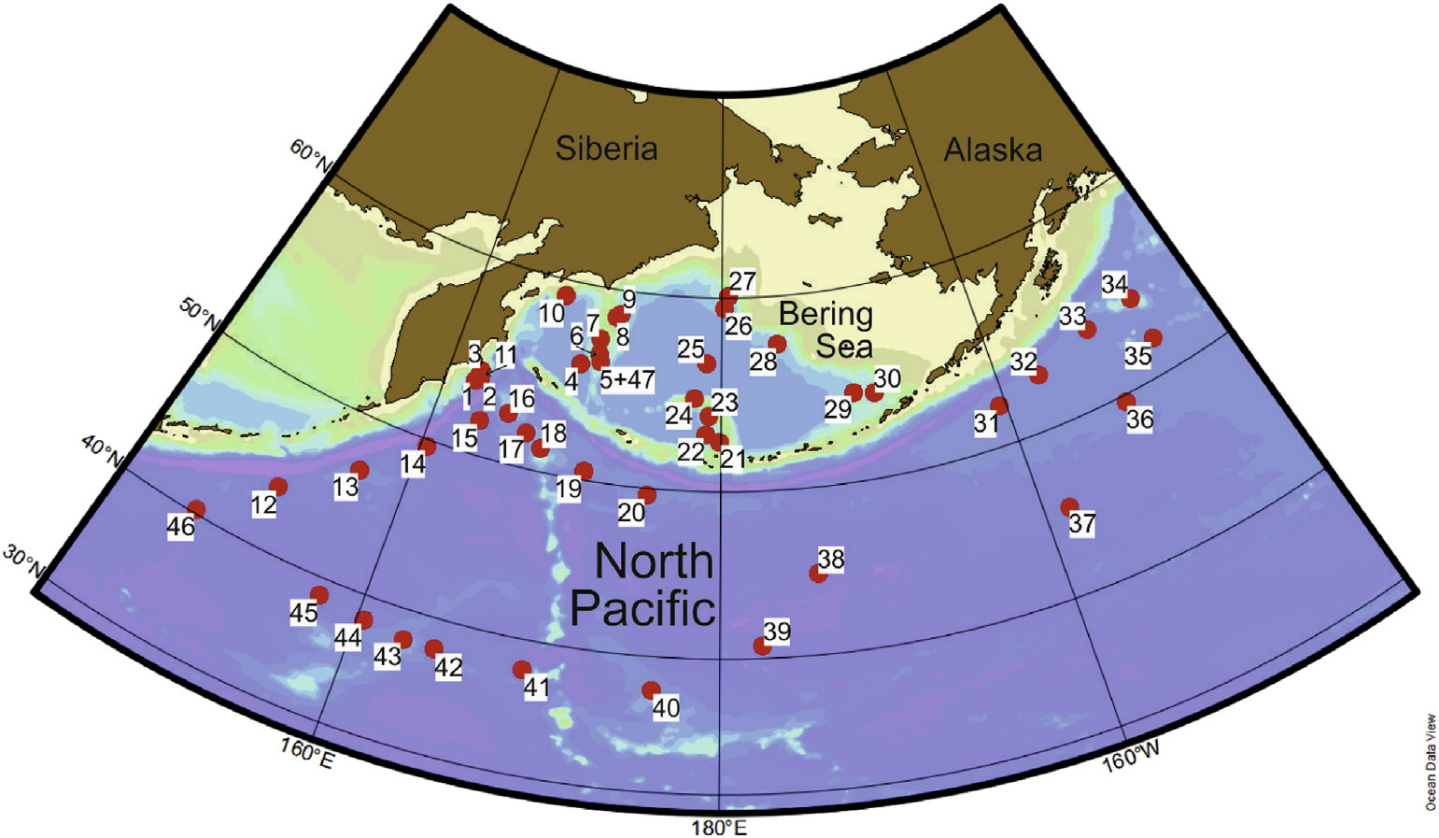
Location of the studied samples. Numbers indicate stations in *Data file 1*.

*Data file 1* presents the coordinates of stations of the KALMAR II and INOPEX projects, RV Sonne SO201-2 and SO202-1 cruises, respectively, where radiolarians were analyzed in the surface sediment samples (Fig. 1). Eleven multicorer stations of KALMAR 2, 35 multicorer stations of INOPEX, and sediment core SO201-2-77KL of KALMAR II are listed. Sediment core SO201-2-77KL is added to the list because some radiolarian slides from this core were used to make the additional microphotographs. Fig. 1 shows the location of stations.

*Data file 2* presents the list of radiolarian taxa, 208 taxa all in all. It contains the microphotographs illustrations of most taxa from the micropaleontological slides of the multicorer surface samples and sediment core SO201-2-77KL.

*Data file 3*. The distribution of all radiolarian taxa (mentioned in *Data file 2*) in the multicorer surface sediment samples (mentioned in *Data file 1*) as (1) raw counts of radiolarian specimens per 1 slide, and (2) calculated total radiolarian abundances per 1 g of dry sediment and taxa percentages.

## 2 Experimental design, materials, and methods

For the radiolarian analysis, we sampled the surface sediments of the upper 0–1 cm layer from every multicorer station listed in *Data file 1*. We used a standard laboratory treatment of the soft marine sediments for cleaning of the biogenic silica tests and preparation of the micropaleontological slides for the microscopic radiolarian analysis developed in AWI [3], [4]. Freeze-dried sediment samples of 0.5–1 g weight were boiled in a solution of the hydrogen peroxide and sodium pyrophosphate. After repeated decantation, sediment residues containing the cleaned radiolarians were sieved through the mesh of 40 μm. The radiolarian tests were randomly settled on the cover slides in the Petri dishes and mounted on the ground slides in the Mountex mounting media.

We counted at least 350–400 radiolarian tests per 1 slide under the Carl Zeiss AxioPlan 2 microscope with a magnification of ×300-600 to ensure a taxonomical identification and calculation of the total radiolarian abundances and species percentages. We tried to identify all radiolarian specimens as species/subspecies or belonging to the higher taxonomical levels like genera, families, subfamilies, etc. An extensive set of the microphotographs of most radiolarian taxa was made.
